# Contrasting Population Structures of the Genes Encoding Ten Leading Vaccine-Candidate Antigens of the Human Malaria Parasite, *Plasmodium falciparum*

**DOI:** 10.1371/journal.pone.0008497

**Published:** 2009-12-30

**Authors:** Alyssa E. Barry, Lee Schultz, Caroline O. Buckee, John C. Reeder

**Affiliations:** 1 Centre for Population Health, Burnet Institute, Melbourne, Australia; 2 Department of Zoology, University of Oxford, Oxford, United Kingdom; 3 Santa Fe Institute, Santa Fe, New Mexico, United States of America; BMSI-A*STAR, Singapore

## Abstract

The extensive diversity of *Plasmodium falciparum* antigens is a major obstacle to a broadly effective malaria vaccine but population genetics has rarely been used to guide vaccine design. We have completed a meta-population genetic analysis of the genes encoding ten leading *P. falciparum* vaccine antigens, including the pre-erythrocytic antigens *csp*, *trap*, *lsa1* and *glurp*; the merozoite antigens *eba175*, *ama1*, *msp*'s 1, 3 and 4, and the gametocyte antigen *pfs48/45*. A total of 4553 antigen sequences were assembled from published data and we estimated the range and distribution of diversity worldwide using traditional population genetics, Bayesian clustering and network analysis. Although a large number of distinct haplotypes were identified for each antigen, they were organized into a limited number of discrete subgroups. While the non-merozoite antigens showed geographically variable levels of diversity and geographic restriction of specific subgroups, the merozoite antigens had high levels of diversity globally, and a worldwide distribution of each subgroup. This shows that the diversity of the non-merozoite antigens is organized by physical or other location-specific barriers to gene flow and that of merozoite antigens by features intrinsic to all populations, one important possibility being the immune response of the human host. We also show that current malaria vaccine formulations are based upon low prevalence haplotypes from a single subgroup and thus may represent only a small proportion of the global parasite population. This study demonstrates significant contrasts in the population structure of *P. falciparum* vaccine candidates that are consistent with the merozoite antigens being under stronger balancing selection than non-merozoite antigens and suggesting that unique approaches to vaccine design will be required. The results of this study also provide a realistic framework for the diversity of these antigens to be incorporated into the design of next-generation malaria vaccines.

## Introduction

Infection with the protozoan parasite *Plasmodium falciparum* causes more than 500 million episodes of clinical malaria and two million deaths each year [1]. A broadly effective malaria vaccine would have a significant global health impact on this enormous public health burden. Over the past 40 years, an intensive international effort has led to the development of several antigens from *P. falciparum* as malaria vaccine candidates. They include surface exposed proteins from morphologically distinct developmental stages of the parasite lifecycle within the human host namely the Circumsporozoite Surface Antigen (CSP), Thrombospondin Related Adhesion Protein (TRAP), Liver Stage Antigen 1 (LSA1), Apical Membrane Antigen 1 (AMA1), Erythrocyte Binding Antigen 175 (EBA175), Merozoite Surface Proteins (MSPs 1–5), Glutamate Rich Protein (GLURP) and Pfs48/45 ([2]; Table 1). Many of these antigens have undergone rigorous developmental and preclinical testing as subunit vaccines [2] but only a few have reached advanced clinical trials (e.g. Phase 2b: CSP (RTS,S); AMA1 (FMP2.1, C1); MSP1_42_ (FMP1); MSP3 (LSP)) [3]. The variable success of candidate malaria vaccines may be due to the high degree of diversity of *P. falciparum* antigens [4] and a variant-specific immune response [5], [6], particularly as most vaccines are formulated with a single polymorphic variant. There is now increasing recognition that a malaria vaccine may need to contain multiple variants of the target antigen to be effective against an entire parasite population [7].

**Table 1:** Summary of population genetic data collected for the genes encoding twelve *P. falciparum* vaccine antigens.

GENE	EXPRESSION*	LOCUS*	DOMAIN	NUCLEOTIDES	*n*	*dN*	*dS*
*csp*	Sporozoite	PFC0210c	C-terminal	909–1140	604	20	3
*trap*	Sporozoite	PF13_0201	N-terminal	1–993	100	70	4
*lsa1*	Liver stages	PF10_0356	N-terminal	1–397	74	12	2
*ama1*	Merozoite	PF11_0344	Region I	448–903	572	46	11
*eba175*	Sporozoite, Merozoite	MAL7P1.176	Region II	433–2169	135	23	2
*msp1*	Merozoite	PFI475w	MSP1_19_	4813–5863	2237	5‡	n.d.
*msp2*	Merozoite	PFB0300c	Blocks 2 & 3	1–816	392	n.d.	n.d.
*msp3*	Merozoite	PF10_0345	Dimorphic repeat†	106–523	124	75	18
*msp4*	Sporozoite, Merozoite	PFB0310c	All	1–816	142	16	3
*msp5*	Merozoite	PFB0305c	All	1–819	70	4	3
*glurp*	Sporozoite, Liver, Blood, Gametocyte	PF10_0344	Region 0	106–1353	48	22	7
*pfs48/45*	Gametocyte	PF13_0247	All	1–1326	55	25	15
**MEDIAN**				817.5	129.5	22.5	3.5
**TOTAL**				10419	4553	313	68

* Source: PlasmoDB, www.plasmodb.org.

† gaps were deleted.

‡ analysis was done only with 5 amino acid polymorphisms, *n* = number of sequences; *dN* = number of nonsynonymous polymorphisms; number of synonymous polymorphisms; n.d. = not done.

The extent and distribution of genetic diversity of *P. falciparum* is for the most part associated with transmission intensity and geographic origin [8], [9], but unique patterns of diversity have been observed for *P. falciparum* antigens. Because many antigens are under immune selection, they are several times more diverse than the neutral loci used in genome wide analyses. This is the case even in low transmission regions [10], [11], [12], suggesting that vaccines will need to represent a large number of variants regardless of the region of deployment. The strong geographic differentiation observed in genome-wide markers [8], [9] is also detectable in genes encoding the sporozoite antigen, *csp* [13], [14], [15] and to a greater extent, the gametocyte antigen, *pfs48/45* [16], raising the possibility that malaria vaccines may need to be tailored for specific regions. However, a lack of geographic differentiation has been observed for blood stage antigens such as *ama1* [17], [18], [19], *msp3* [20], *msp4,5* [21] and *S-antigen* [22]. *Ama1* variants have recently been shown to cluster into six genetically distinct subgroups on the basis of antibody cross-reactivity, with all subgroups being found worldwide. This study illustrated that immune selection may play a role in structuring the diversity of this highly polymorphic antigen. Consequently, a small number of variants from distinct subgroups may give the sought after broad vaccine coverage [18]. To inform the design of next generation malaria vaccines, population genetic studies for each candidate antigen in the spectrum of endemic regions will be essential. Such analyses will help to prioritize candidates, advance our understanding of the geographic distribution of genetic diversity and provide a framework for testing the immunological significance of antigen diversity.

An enormous amount of research has highlighted the extensive diversity of *P. falciparum* antigens [23], however the majority of studies have focused on just one or two countries per antigen and comparisons among studies have rarely taken place. To facilitate the design of broad-spectrum malaria vaccines, we have summarized the known global range and distribution of genetic diversity of ten leading malaria vaccine antigens for which population-level sequence data was available. We collected sequences from natural populations and laboratory-isolates and completed a population genetic analysis using a variety of traditional and more recently developed clustering tools. By comparative analyses we show evidence that the diversity of non-merozoite antigens is largely structured on the basis of geographic origin while for merozoite antigens, the dominant targets of natural host immunity [24] a relative lack of geographic structure was observed with the majority of diversity being contained within each location. This meta-population genetic analysis of ten leading malaria vaccine candidates provides a framework by which to consider parasite diversity in the design of the next generation of malaria vaccines.

## Results

### Data summary

More than 4500 sequences with an average length of 0.8 kb were compiled from GenBank and the published literature for the genes encoding twelve antigens that matched the inclusion criteria (Tables 1, S1 and S2). Although *msp2* and *msp5* matched the criteria we did not complete the population genetic analyses. For *msp2*, this was due to the majority of sequences being comprised of highly polymorphic repeats (with many gaps) flanked by only short regions of unique sequence. Haplotypes could therefore only be defined on the basis of differing numbers of repeats, resulting in an overestimation of biologically significant diversity. For *msp5*, there were only five haplotypes and preliminary analyses showed that they were not structured within nor among populations (data not shown), so diversity in this antigen was also unlikely to have major biological significance. Among the remaining ten antigens, the number of nonsynonymous polymorphisms (*dN*) was several-fold greater than the number of synonymous polymorphisms (*dS*; Table 1), which is an indication of immune selection in the *P. falciparum* genome [4]. The population dataset included sequences from the natural parasite populations of between 2 and 13 countries and a minimum of 2 geographical regions (namely Americas (Central and South), Asia Pacific or Africa, Table S1). The median sample size was 31 sequences (range = 8–1368) per country, and each country contained a median number of 8 distinct haplotypes (range = 1–68) (Table 2). Only small sample sizes were available for *glurp* and *pfs48/45* so we caution that the results for these antigens may be biased and thus should be interpreted with care. To focus the analysis on the putative antigenic diversity (i.e. polymorphisms that change protein structure) the nonsynonymous single nucleotide polymorphism (nsSNP) haplotypes were derived for all antigen sequences, except for *msp1*, for which the majority of the data comprised only a 5 amino acid haplotype (corresponding to polymorphisms found only in the MSP1_19_ domain), so the remaining *msp1* DNA sequences were converted to the corresponding amino acid haplotype. It is important to note that haplotypes are simple combinations of nucleotides or amino acids with no particular weight placed upon any position or change, rather all of the following analyses were based on whether each polymorphic site was the same or different.

**Table 2:** Estimates of diversity for the genes encoding ten *P. falciparum* vaccine antigens.

GENE	REGION	COUNTRY	*n*	*S*	*k*	*π* (×10^−3^)	*h*	*Hd*
*csp*	Americas	Brazil	31	5	1.35	5.85	3	0.28
		Venezuela	10	13	6.24	27.9	6	0.89
	Asia Pacific	Vanuatu	136	2	0.62	2.7	2	0.31
		Indonesia	36	8	0.65	2.83	5	0.26
		Vietnam	143	14	2.06	8.9	20	0.7
		Thailand	26	13	2.95	12.76	8	0.76
		Myanmar	25	6	1.08	4.68	4	0.41
		India	11	2	0.8	4.25	3	0.47
		Iran	91	3	1.08	4.68	5	0.6
	Africa	Kenya	18	17	5.73	25.29	13	0.93
		Cameroon	9	12	4.56	19.72	7	0.94
		The Gambia	44	18	5.97	25.83	21	0.95
		Senegal	10	10	4.73	20.49	8	0.96
*trap*	Asia Pacific	Thailand	29	22	5.6	6.12	25	0.99
		India	8	30	9.89	10.86	8	1
	Africa	The Gambia	48	46	11.58	12.13	37	0.98
*lsa1*	Americas	Brazil	19	8	1.81	4.83	6	0.7
	Asia Pacific	Papua New Guinea	20	7	2.31	6.07	7	0.88
		Malaysia	10	3	1.02	3.97	3	0.51
	Africa	Kenya	22	8	2.18	6.12	7	0.83
*ama1*	Americas	Venezuela	10	19	7.27	18.71	6	0.78
	Asia Pacific	Papua New Guinea	162	34	11.11	26.3	27	0.94
		Thailand	55	30	10.75	25.5	19	0.94
		India	101	44	9.72	24.31	68	0.99
	Africa	Kenya	8	24	9.54	24.2	8	1
		Nigeria	51	34	11.39	27.14	35	0.98
		Mali	61	37	11.1	26.97	40	0.98
		Benin	22	30	9.99	25.08	20	0.99
*eba175*	Asia Pacific	Thailand	48	18	6.23	3.81	17	0.9
	Africa	Kenya	39	18	6.01	3.47	23	0.9
		Nigeria	30	16	5.53	3.19	15	0.81
*msp1*	Americas	Brazil	138	n.a.	n.a.	n.a.	7	0.71
		Peru	135	n.a.	n.a.	n.a.	1	0
	Asia Pacific	Solomon Is.	77	n.a.	n.a.	n.a.	4	0.61
		Vanuatu	140	n.a.	n.a.	n.a.	3	0.61
		Phillippines	57	n.a.	n.a.	n.a.	5	0.74
		Vietnam	77	n.a.	n.a.	n.a.	5	0.56
		Thailand	72	n.a.	n.a.	n.a.	5	0.64
		India	51	n.a.	n.a.	n.a.	10	0.83
		Iran	92	n.a.	n.a.	n.a.	5	0.8
	Africa	Kenya	18	n.a.	n.a.	n.a.	6	0.77
		Mali	1368	n.a.	n.a.	n.a.	15	0.76
*msp3*	Asia Pacific	Thailand	50	75	27.94	96.62	9	0.71
	Africa	Nigeria	51	86	30.33	106.63	12	0.81
*msp4*	Asia Pacific	Papua New Guinea	42	9	2.42	3.1	14	0.92
		Cambodia	12	9	2.74	3.36	9	0.94
		Thailand	15	9	2.8	3.43	10	0.93
	Africa	Senegal	41	15	2.88	3.87	23	0.95
*glurp*	Americas	Brazil	9	9	4.78	3.85	5	0.72
	Asia Pacific	Myanmar	10	9	3.2	2.58	9	0.98
	Africa	Senegal	11	1	0.18	0.15	2	0.18
*pfs48/45*	Americas	Venezuela	9	12	2.94	3.06	6	0.83
	Asia Pacific	Thailand	10	4	0.8	0.6	2	0.2
		India	10	8	1.91	1.59	4	0.53
	Africa	Kenya	15	11	2.67	3.94	8	0.88

*n* = number of sequences, *S* = number of variant sites, *h* = number of haplotypes, *Hd* = haplotype diversity, *k* = average number of pairwise differences, *∏* = nucleotide diversity.

### Polymorphism and haplotype diversity

**Figure 1:**
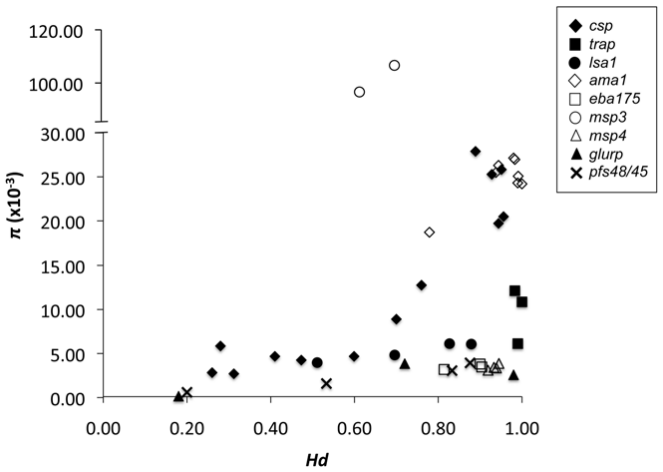
The relationship between polymorphism and haplotype diversity of the genes encoding ten *P. falciparum* vaccine antigens. Non-merozoite antigens are represented by a solid symbol and merozoite antigens by an open symbol.

Comparing among countries for each antigen, the genes encoding the non-merozoite antigens (*csp*, *trap*, *lsa1*, *glurp* and *pfs48/45*; Table 1) showed variation in diversity as measured by the polymorphism (*k* and *∏*) and haplotype diversity (*Hd*) statistics. Whereas, the genes encoding each of the merozoite antigens (*ama1*, *eba175* and *msp1*, *msp3* and *msp4*; Table 1) each showed similar levels of diversity among countries and regions (Table 2). For example, *csp* was significantly more diverse in African compared to Asia-Pacific countries (*P*<0.01) while for *ama1* there were no significant differences between African and Asia-Pacific countries (*P*>0.05). Furthermore, for non-merozoite antigens the degree of haplotype diversity was strongly correlated with the amount of polymorphism (*ρ* = 0.63, *P*<0.01), whereas for the merozoite antigens, the amount of polymorphism (*k* and *∏*) varied widely among antigens but *Hd* was almost always high (Figure 1; Table 2; *ρ* = 0.13; *P*>0.05). Therefore, haplotype diversity varied widely among countries and regions for non-merozoite antigens in association with transmission intensity and polymorphism, but was consistently high for the merozoite antigens irrespective of transmission intensity and levels of polymorphism suggesting that the latter are under stronger balancing selection.

### Genetic differentiation and gene flow

To determine how the observed diversity was distributed among countries, population structure was first inferred by measuring genetic differentiation among countries both within and among regions. To do this we calculated *F*_ST_ from haplotype-frequencies and pairwise DNA sequence diversity, although only the former was calculated for MSP1 (see Materials and Methods). The haplotype frequency-based statistics are more sensitive for small sample sizes, while the sequence based statistic is a more sensitive method for detecting population structure in highly polymorphic loci [25], (see Text S1 for specific examples). Significant differentiation was identified among regions for all *P. falciparum* vaccine antigens, albeit to a lesser degree for *lsa1*, *ama1*, *eba175* and *msp4* compared to the other 6 antigens (Table 3A,B). This regional differentiation was accompanied by limited gene flow (*Nm*) for all antigens except for low-moderate levels for *lsa1*, *ama1* and *eba175* and very high for *msp4* (Table 3C). Differentiation was also detected within the Americas for *csp* and *msp1* (the only antigens for which we had multiple populations within this region), within the Asia Pacific for *csp*, *trap*, *ama1 and msp1*, and significant but low levels of differentiation within Africa for *ama1* (Table 3). For MSP1, the Asia-Pacific countries spanned a broad area. Accordingly, pairwise comparisons identified differentiation between the Pacific and mainland Asian countries (*F*_ST_ = 0.06–0.31; *P*<0.01). Significant differentiation was also observed in pairwise comparisons of countries from East (Vietnam, Thailand) and West (India, Iran) mainland Asia (*F*_ST_ = 0.09–0.27; P<0.001) with no structure among countries within these subregions (*F*_ST_ = 0, *P*>0.05; 0.03, *P*<0.05 respectively). Additional structure was detected among the Pacific island nations (*F*_ST_ = 0.07–0.21; *P*<0.01). We also observed significant differentiation between local populations such as Vanuatu's islands for *csp* (Pentecost compared to Gaua: *F*_ST_ = 0.14; *P* = 0.02; and Malakula: *F*_ST_ = 0.19; *P*<0.01) and MSP1 (all comparisons, *F*_ST_ = 0.17–0.54; *P*<0.01) and distant locations of India for MSP1 (*F*_ST_ = 0.32; P<0.001). No such structuring was observed for *csp* within Brazil or Myanmar, *ama1* in PNG or Thailand, *msp1* in Vietnam, nor *msp4* in Senegal (Table S1).

**Table 3:** Estimates of genetic differentiation and gene flow for the genes encoding ten *P. falciparum* vaccine antigens.

	AMONG COUNTRIES			AMONG REGIONS
	AFRICA	ASIA PACIFIC	AMERICAS	
**A.**				
*csp*	0.02**	0.08***	0.27***	0.21***
*trap*	n.a.	0.0021	n.a.	0.007**
*lsa1*	n.a.	0.05*	n.a.	0.09***
*ama1*	<0.01	0.02***	n.a.	0.03***
*eba175*	0.01	n.a.	n.a.	0.05***
*msp1*	0.03	0.18***	0.42***	0.22***
*msp3*	n.a.	n.a.	n.a.	0.07***
*msp4*	n.a.	0.01	n.a.	0.02**
*glurp*	n.a.	n.a.	n.a.	0.30***
*pfs48/45*	n.a.	0.03	n.a.	0.20***
**B.**				
*csp*	0.02	0.08***	0.29***	0.25***
*trap*	n.a.	0.12***	n.a.	0.12***
*lsa1*	n.a.	0.03ns	n.a.	0.09**
*ama1*	0.01*	0.02***	n.a.	0.03***
*eba175*	<0	n.a.	n.a.	0.07***
*msp1*	n.a.	n.a.	n.a.	n.a.
*msp3*	n.a.	n.a.	n.a.	0.11***
*msp4*	n.a.	0.01	n.a.	0.02*
*glurp*	n.a.	n.a.	n.a.	0.40***
*pfs48/45*	n.a.	<0	n.a.	0.22***
**C.**				
*csp*	10.56	2.24	0.37	0.70
*trap*	n.a.	0.30	n.a.	0.40
*lsa1*	n.a.	3.37	n.a.	1.76
*ama1*	6.63	8.34	n.a.	3.36
*eba175*	−39.11	n.a.	n.a.	2.03
*msp1*	8.08	1.14	0.35	0.89
*msp3*	n.a.	n.a.	n.a.	0.85
*msp4*	n.a.	−22.24	n.a.	38.48
*glurp*	n.a.	n.a.	n.a.	0.18
*pfs48/45*	n.a.	−4.82	n.a.	0.65

* 0.01<*P*<0.05.

** 0.001<*P*<0.01; *P*<0.001; n.a. not applicable.

For each antigen, *F*_ST_ statistics were calculated from both (A) haplotype frequencies and (B) sequence diversity (except for MSP1 for which only haplotype frequencies were used) and (C) gene flow (*Nm*). *Nm*>1 is considered a high level of gene flow. The *P*-values shown in the key are for *F*_ST_ only and were not available for Nm.

### Clustering and networks

**Figure 2:**
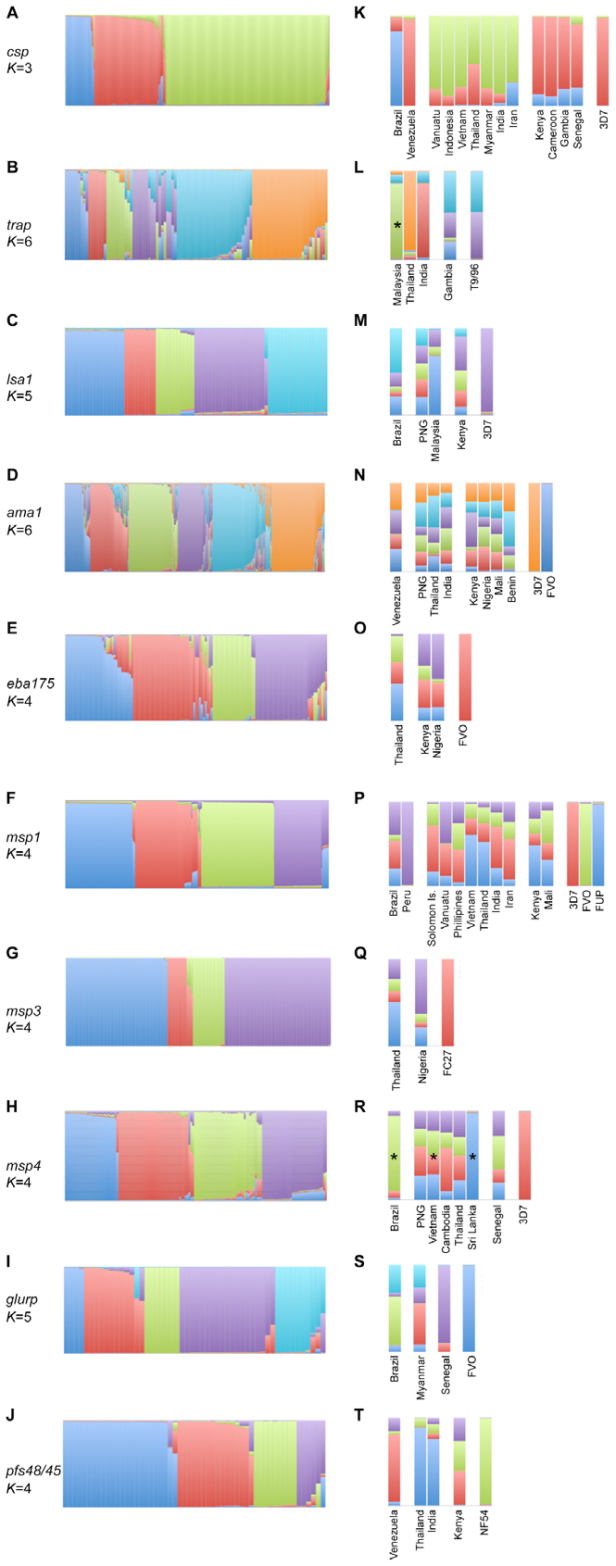
Global population structure of the genes encoding ten *P. falciparum* vaccine antigens based on Bayesian cluster analysis. Membership coefficients for A–J) individual nsSNP haplotypes and K–T) the population average for the estimated number of clusters (*K*, shown on the left of the two histograms). In the latter, countries from different continents are separated by a blank space and organised from east on the left, to west on the right with vaccine haplotypes on the far right hand side. An asterisk denotes countries for which fewer than 8 haplotypes were available that were taken from dataset 2 (Table S2). Dark blue = cluster 1; Red = cluster 2; Green = cluster 3; Purple = cluster 4; Light blue = cluster 5; Orange = cluster 6.

In the analysis so far, individuals were grouped by geographic location, assuming that geography (or other associated variables e.g. host genetics, vector species) will be the dominant barrier to gene flow. It is possible that these somewhat arbitrary groupings might incorrectly estimate population structure, or fail to identify within population subdivision, although where possible, we measured genetic differentiation within a country as described above (Table S1). To address this, and to identify subgroups of related nsSNP (or for MSP1, amino acid) haplotypes that are genetically and thus potentially antigenically distinct, we also used a Bayesian clustering algorithm [26], [27] and confirmed the results using network analysis (see Materials and Methods). The Bayesian algorithm groups related haplotypes into a predefined number of clusters (*K*) on the basis of shared allele frequencies. Each haplotype is then assigned a membership coefficient (*Q*) to each of the clusters with the majority of the haplotypes being assigned to only one cluster at “true” *K* (Figure 2 A–J) and variability in the data increasing thereafter (Figure S1; [26], [27]). Using this approach we found a small number of distinct clusters for all antigens (*K*_mean_ = 4.5, *K*_range_ = 3–6). Although admixed haplotypes (<75% membership to any one cluster) were prevalent for *trap*, *ama1*, *eba175* and *msp4* (Figure 2 B, D, E and H), increased estimates of *K* resulted in even higher proportions of admixed haplotypes (Figure S2) thus confirming that the distribution of the haplotypes was best explained by the *K* presented in Figure 2 (A–J). Network analysis differs in that it simply shows connectivity among all haplotypes on the basis of shared SNPs and allows for the visualization of recombinant haplotypes that bridge the major subgroups. If haplotypes differed by fewer nsSNPs than the predefined threshold (*t*), they were connected, and if greater than *t* they were not. We used a *t*-value that connected the majority of haplotypes so all relationships could be examined in one network, and for clarity. The results supported the cluster analysis with haplotypes grouping into a small number of tightly connected lobes that corresponded to each of the *structure* defined subgroups (Figure S3). Bridging connections were predominantly characterized by admixed haplotypes or entire subgroups (e.g. *ama1*, *msp4*) as defined by the cluster analysis and suggest that these comprise recombinant haplotypes (Figure S3).

**Figure 3:**
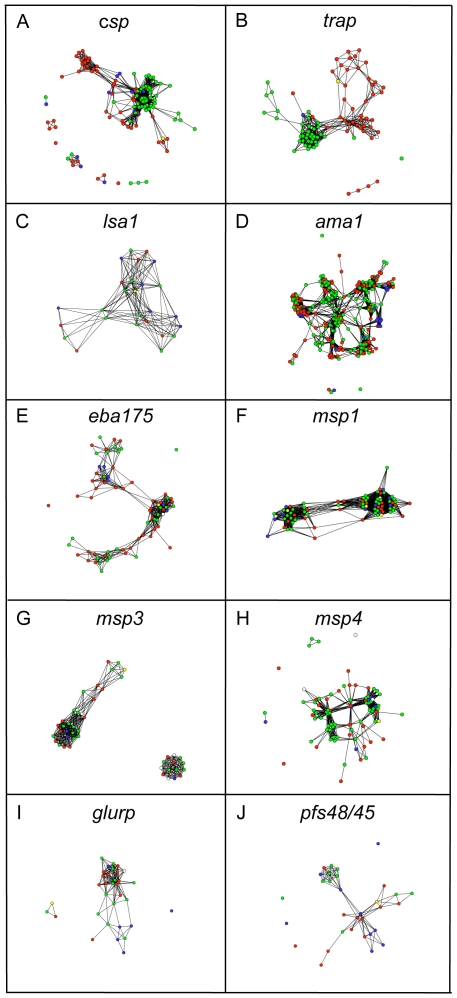
Global population structure of the genes encoding ten *P. falciparum* vaccine antigens based on network analysis. Networks of nsSNP haplotypes were drawn by first removing multiple copies of each haplotype , leaving only one copy per country for the analysis. Hence, identical haplotypes from different regions, but not within regions were included. Each node (coloured circle) represents a haplotype, shaded by region of origin: Red = Africa, Green = Asia, Blue = Americas. Nodes are tied by edges (black lines) demonstrating that they share a predefined threshold (*t*) of nsSNPs for *csp* = 24; *trap* = 67; *lsa1* = 8; *ama1* = 48; *eba175* = 18; *msp1* = 5; *msp3* = 63; *msp4* = 19; *glurp* = 18; *pfs48/45* = 23. Vaccine haplotypes are shaded in yellow. Haplotypes originating from isolates with unknown origin are shaded in white (unless they were vaccine haplotypes).

To determine whether the above-defined “subgroups” were geographically restricted, for the Bayesian cluster data we plotted the average *Q* for each country (Figure 2 K–T), and calculated the average frequency of haplotypes with membership to the predominant subgroup (*f_m_*) and the population diversity (*Pd*) a simple measure of the distribution of clusters that is analogous to the *Hd* (see above and Materials and Methods). Globally (i.e. comparisons among all countries), the cluster analysis supported the high levels of differentiation among regions for the non-merozoite antigens with a high frequency of haplotypes belonging to one subgroup (*f_m_* = 69.5±8%) and low population diversity (*Pd* = 0.40±0.07), albeit *lsa1* showed low to medium frequencies of all clusters in PNG (*Pd* = 0.80) and Kenya (*Pd* = 0.74; Figure 2 M; Table S3). In contrast, all of the merozoite antigens showed low to medium frequencies of all clusters in all countries (*f_m_* = 45.8±7%) and high population diversity (*Pd* = 0.64±0.07), although the frequency of each cluster was variable among countries (Figure 2 N–R; Table S3). These variations in frequency were consistent with the moderate geographic differentiation described above. The network analysis further supported these results with the non-merozoite antigen haplotypes being most strongly connected with others originating from the same geographic region (Figure 3 A–C, I, J), whereas for the merozoite antigens, haplotypes from different regions often connected within the same lobes of the network (Figure 3 D–H). These analyses also supported the diversity analyses with (for example) the highly diverse African *csp* and *trap* haplotypes being loosely or disconnected from the main network (Figure 3 A, B), whereas geographic origin did not correlate with the connectivity of the ubiquitously diverse merozoite antigen haplotypes to the network (Figure 3 D–H).

Within regions, the significant differentiation detected in the Americas for both *csp* and MSP1 (Table 3), was supported by the cluster analysis (note that network analysis is not presented at this or any finer resolution). For *csp* the majority of haplotypes showed membership to one cluster, albeit different clusters for each country (Figure 2K; Table S3). For MSP1, a single cluster was found in Peru (1 haplotype, Table 2) and 4 clusters (7 haplotypes; including that found in Peru) were found in Brazil (Figure 2P; Table S3). In the Asia-Pacific region, for *trap* the differentiation between Thailand and India was supported by the cluster analysis (Figure 2L; Table S3). Whereas, for *csp*, *lsa1*, *ama1* and MSP1 varying degrees of support were given to the differentiation observed with the same clusters being found in all Asia Pacific countries albeit at variable frequencies (Figure 2 K, M, N and P). For *csp*, a single cluster was predominant among all Asia-Pacific countries, with low frequencies of haplotypes belonging to a second cluster. The majority of Iranian haplotypes clustered with the Asia-Pacific cluster and a minor proportion with the African cluster, a structure that is consistent with Iran's central location between these two regions. For *lsa1*, all clusters were found in PNG, but only 2 in Malaysia. A limited degree of differentiation between the two countries (Table 3), and lower diversity in Malaysia (Table 2) suggests that it shares haplotypes with PNG. For *ama1*, all clusters were present in each country at variable frequencies and high population diversity (Figure 2N, 3). This is consistent with a previous report [18], albeit our dataset contained 3.6 times the number of haplotypes (Table 1). For MSP1, the differentiation seen among Asia-Pacific subregions was supported by variable frequencies of the four clusters (Figure 2 P; Table S3). The remaining two antigens studied in the Asia Pacific, *msp4* and *pfs48/45*, showed strong similarities in the cluster analysis (Figure 2 R, T) demonstrating a lack of population structure for these antigens in this region. Among African countries, the cluster analysis confirmed a lack of population structure with strong similarities among countries for the four antigens for which multiple African sites were sampled (Figure 2 K, N–P). To identify local population structure, we investigated differences among locations within the same country. The results show that only MSP1 and *pfs48/45* were structured among locales within a country (Figure S4).

### Prevalence of vaccine haplotypes

The majority of haplotypes upon which current vaccines are based were found to be present, but at extremely low frequencies in the global parasite population, with higher frequencies observed only for *lsa1* and MSP1 vaccine haplotypes (Table 4). Because distinct haplotypes may be different by as few as one nsSNP, which is less likely to encode antigenic differences than multiple nsSNPs, the breadth of biologically significant similarities may be underestimated by this analysis. Therefore, we also included vaccine haplotypes in the cluster (Figure 2 K–T) and network analysis (Figure 3) to identify their associated subgroups. MSP1 vaccine haplotypes grouped with three distinct subgroups of the four defined (Figure 2 P; Figure S3 F), each representing the most common haplotypes. For the remaining antigens, the subgroups to which vaccine haplotypes associated were of a limited prevalence in all populations (*ama1*, *eba175*, *msp3*, *msp4*, *glurp*, *pfs48/45*) (Figure 2) or were geographically restricted (*csp*, *trap*, *lsa1*) (Figure 2, 3). All of the laboratory isolates (Table S2) were also included in the cluster and network analysis. This allowed the assignment of these isolates to haplotypes and *structure* defined clusters, thus providing a framework for experiments to test the biological significance of diversity and identifying the most distinct haplotypes for diversity-covering vaccines (Table S4; [18]).

**Table 4:** Worldwide prevalence of *P. falciparum* antigen haplotypes that are components of malaria vaccines.

GENE	VACCINE*	HAPLOTYPE	PREVALENCE
*csp*	3D7	1	0.01
*trap*	T9/96	59	0.01
*lsa1*	3D7	1	0.23
*ama1*	3D7, FVO	2, 3	0.06, 0.06
*eba175*	FVO	2	0.13
*msp1*	3D7, FVO, FUP	1, 2, 3	0.16, 0.28, 0.26
*msp3*	FC27	6	0.10
*msp4*	3D7	1	0.13
*glurp*	FVO	8	0.06
*pfs48/45*	NF54	11	0.09

* Names of laboratory isolates used for vaccine development.

## Discussion

To provide a rational framework for incorporating diversity into the next generation of malaria vaccines, we have completed a meta-population genetic analysis and thus summarised the known global range and natural distribution of diversity for ten leading malaria vaccine candidates. There are many natural population datasets available from previous studies and there is a strong precedent for comparing multiple datasets for such studies even when only small numbers of samples are available (e.g. [17], [28]). Sample size was a limitation for the population genetic analysis of some antigens and locations, however the majority of natural populations (>70%) were represented by at least 20 sequences. For populations with less than this number of sequences the results should be interpreted with care. Despite these small sample sizes, similar results to other countries from the same region were observed. For example, Indian *csp* sequences (n = 11) showed a similar pattern of diversity to other Asian countries with larger sample sizes (n = 25–143), as did Thai and Indian *pfs48/45* sequences (n = 10 for both). We also used haplotype frequencies to measure differentiation, which has been shown to be more reliable than sequence diversity for smaller sample sizes [25] but results for both statistics were similar for antigens with smaller sample sizes (*lsa1*, *glurp*, *pfs48/45*). The patterns of diversity and geographic population structure observed for these antigens warrant further investigation by deep sampling in each geographic region. Another potential source of bias is the combination of data from different time points and from patients with different clinical status (Table S1). Frequency dependant selection acts on antigens under strong immune selection [29] resulting in changes in allele frequency over time, and thus may exaggerate differentiation or alter cluster frequencies seen among countries within the same region, such as that observed for *ama1* and MSP1 in the Asia-Pacific. Clinical samples may also be biased toward particular antigen haplotypes [30], [31], [32], [33]. Nevertheless the differences among countries (particularly evident in the large Asia-Pacific region) appeared to increase with geographic distance, independently of both time and clinical definition (Table S1), so these factors should not change the overall conclusions of this study. A phenomenal amount of additional sampling and sequencing, requiring a vastly inflated budget and a major international consortium would be needed to address these sampling issues. Our strategy, in using population genetic data already generated and freely available has revealed important insights into the overall organization of genetic diversity of vaccine antigens and provides a framework for future studies to improve malaria vaccine design.

By comparing the diversity found in different countries worldwide we demonstrated that *csp*, and to a lesser extent *trap* and *lsa1* showed similar patterns to that of putatively neutrally evolving microsatellite and SNP markers [8], [34], [35]. The highest levels of diversity were found in Africa where transmission is holoendemic (very high), the lowest in the Americas where it is hypoendemic (low) and moderate levels in the Asia-Pacific where transmission ranges from meso-hyperendemic (medium to high). This suggests that transmission plays a predominant role in the diversification of these non-merozoite antigens, and the similarities to neutral markers suggest that these genes are not under strong balancing selection. *Glurp* and *pfs48/45* also showed similarly variable diversity but there was no apparent trend for higher diversity in Africa compared to other regions and as mentioned above the small sample size for these antigens makes it difficult to draw solid conclusions. For the merozoite antigens, the observation of high levels of haplotype diversity among countries at different ends of transmission spectrum even for antigens with low levels of polymorphism (e.g. *eba175*, *msp4*) suggests that recombination generates a number of different haplotypes even where significant functional constraints exist. Together with immunological evidence that blood stage antigens are major targets of natural host immunity [24], this is a strong indication of balancing (e.g. immune) selection. Immune selection favours a low-medium frequency of distinct haplotypes and thus increased probability of newly infecting parasites carrying antigenically distinct haplotypes to those previously encountered by the host. Therefore, if vaccine candidates are prioritized on the basis of low levels of polymorphism, careful consideration must also be given to distribution of haplotypes within natural populations.

A successful malaria vaccine will need to target a large proportion of the parasite population, but it would not be feasible to vaccinate individuals with the large numbers of haplotypes we have described. A single haplotype will have some capacity to elicit cross-reactive responses against those that are *genetically* similar but the exact amount of polymorphism that defines *antigenically* different haplotypes is not well understood. Recent work has shown that *ama1* haplotypes were organized into six strongly differentiated subgroups by the Bayesian algorithm implemented in the program *structure* [27], [36]. In this study, evidence from invasion inhibition assays suggested that haplotypes from the same subgroup were antigenically similar and thus able to elicit cross-reactive antibody responses, whilst those from different subgroups were antigenically distinct [18]. Therefore, clustering tools may be useful in defining biologically significant variation in *P. falciparum* antigens. Our analysis used two different clustering tools to subgroup the compiled haplotypes, namely the Bayesian clustering and network analysis. Our dataset contained a much larger number of *ama1* sequences (n = 572, compared to 158 in the previous study [18]), with several additional natural populations, yet did not identify any further subgroups. By completing these analyses for all of the leading vaccine antigens in our study we found as few as three, and no more than six subgroups for any antigen in the worldwide parasite population. This suggests that for all ten of the leading vaccine antigens, it may be feasible to cover diversity by inclusion of a small number of carefully selected haplotypes from each subgroup. However, a large number of admixed haplotypes in the cluster analyses or bridging connections among major lobes in the network analyses indicates recombination occurs among subgroups and that there is potential for the evolution of further antigenically distinct haplotypes. Notably, three of the four antigens for which these putative recombinants were common were merozoite antigens (*ama1*, *eba175* and *msp4*). A series of experiments now needs to be done for each antigen to verify the immunological relevance of the patterns observed, the haplotypes from each subgroup that will elicit broadly protective immune responses, and to quantify the contribution of each polymorphism to antigenic diversity.

The geographic distribution of the defined diversity must also be a consideration in the design of a broad-spectrum malaria vaccine because significant variation among regions would suggest a need for vaccines to be tailored accordingly. When we investigated the geographic distribution of diversity for each of the ten vaccine antigens we found stark contrasts among antigens from the different developmental stages of the parasite lifecycle. Although tests of genetic differentiation and gene flow among countries suggested among-region structuring of diversity for all antigens, stronger differentiation among countries and/or regions was found for the non-merozoite antigens. The cluster and network analyses supported strong among region structure (and lower within location diversity) for *csp*, *pfs48/45* and *glurp* and that within regions for *trap* and *lsa1*, albeit much weaker geographic structuring for the latter antigen. By contrast, the merozoite antigens generally had lower levels of among and within region differentiation and gene flow, and haplotypes formed subgroups independent of geographic origin with uniformly high levels of within population diversity. These comparative analyses confirm that there are extreme differences in the population structure of different types of antigens and thus may explain why paradoxical estimates of the most recent common ancestor of *P. falciparum* have been obtained in the past by evolutionary biologists using these markers (reviewed in [37]). Interestingly, the cluster analyses also showed differing frequencies of shared subgroups among countries, which were previously shown to vary over time for *ama1* [18]. This may reflect both geographic isolation and natural fluctuations over time as a result of frequency dependent selection or may simply be the result of the variable sample collection mentioned above. For MSP1, strong differentiation and a variable cluster frequency among sub-regions and island nations of the Asia-Pacific suggests that the biogeography of this region constitutes a strong barrier to gene flow. If the subgrouping of haplotypes is immunologically significant, current vaccine formulations may only target parasites carrying haplotypes from the same subgroup, giving those carrying haplotypes from distinct subgroups a selective advantage. To give a greater probability of broad efficacy, a population-specific vaccine strategy incorporating haplotypes representative for the region may be effective for the non-merozoite antigens while a diversity-covering approach may be necessary for the merozoite antigens.

There are a number of possible explanations for the contrasting population structures of *P. falciparum* antigens. The stronger geographic population structure observed in the non-merozoite compared to the merozoite antigens may at least in part driven by the biology and kinetics of the lifecycle, with shorter, less frequent exposures to human immunity. Therefore, a background of geographic barriers or other location-specific environmental factors will shift the distribution of diversity *among* populations. This is a possibility for *csp* and *trap* which are expressed on the surface of a small number of sporozoites (∼20 parasites) that rapidly migrate to the liver after inoculation into the human host by the mosquito and *pfs48/45* which is expressed only in the mosquito stages [38], [39], [40], [41], [42]. Similarly, *lsa1* is expressed by liver schizonts but is a strong target of naturally acquired immunity [39], [43], [44], in agreement with the weaker geographic structuring of this antigen. *Glurp* is unusual because it is expressed in a number of stages exposed to the human immune response including on the sporozoite, liver schizont, merozoite and gametocyte [38] and shows very strong geographic structuring, however it is possible that the small sample size for each population has overemphasised the diversity among locales. Other region-specific factors that may decrease gene flow among *P. falciparum* populations include human genetic polymorphisms that confer resistance to malaria [45] and adaptation to different anophelene species that transmit *P. falciparum* worldwide [46]. These “bottlenecks” may lead to population structure in genes expressed during the human or mosquito stages respectively, and in neutral loci as markers of the underlying population biology [8], [35]. For the merozoite antigens the diversity *within* populations may be high as a result of exposure to the host immune response. These antigens are all exclusively expressed during the merozoite stage except for *eba175* and *msp4*, which are also expressed in the sporozoite [47], [48], [49]. Merozoite exposure is brief (<2 mins), but it occurs repeatedly at a high parasitemia (>10,000 parasites in the first cycle, thereafter increasing exponentially) so there are many opportunities for immune selection. Some diversification of merozoite antigens may be adaptations to polymorphisms in erythrocyte receptors essential for parasite invasion [50]. Finally, antigens from both groups that are expressed in the mosquito stages (i.e. *csp*, *trap*, *eba175*, *msp4*, *glurp* and *pfs48/45*) may be exposed to immune selection by the anophelene vector (e.g. *csp* [14]). In support of the biological significance of the contrasting population structures observed, balancing selection has been detected in all of the merozoite antigens [51], [52], [53], [54], [55] whereas for the non-merozoite antigens, balancing selection was detected in *trap* and *pfs48/45* [56], [57] but not in *csp* [57] and *lsa1* [58] (*glurp* has not been investigated). Furthermore, a vaccine-mediated haplotype-specific immune response was detected for recombinant vaccines based upon *msp1* [59] and *msp2* [6] but not for *csp* [60], [61] suggesting that different haplotypes are antigenically distinct for the former two antigens. The results of our study are consistent with the structuring of diversity by balancing selection for the merozoite but not for the non-merozoite antigens.

This investigation has revealed a possible framework by which to formulate malaria vaccines with a greater potential for broad protection against the enormous diversity of parasite antigens. It may be possible to tackle the neglected problem of antigen diversity in malaria vaccine design by inclusion of the most prevalent haplotype(s), or a diversity-covering vaccine with inclusion of at least one representative haplotype from each of the defined subgroups of haplotypes. Because they show different population structures, the former approach may be more appropriate for the non-merozoite antigens, and the latter for the merozoite antigens. The haplotype and subgroup classification for a number of laboratory isolates are available in the supporting online material (Table S4) as a first step to guide the selection of such haplotypes, and to help define immunological correlates of protection which are now urgently needed to support these important findings. Nevertheless, if these contrasting population genetic structures of the genes encoding *P. falciparum* antigens are considered in the design of next generation vaccines, perhaps the best test of biological relevance will be the outcome of the ensuing vaccine trials.

## Materials and Methods

### Data collection

The *P. falciparum* antigens selected for the study were key components of malaria vaccines in the late stages of development or in recent trials [2], [3]. To be included in the study, we searched for population data - which we defined as 8 or more sequences from a defined location (e.g. a village or town) - for a minimum of two countries for each antigen. DNA sequences (and amino acid polymorphisms for MSP1) were then obtained for the twelve antigens meeting these criteria, including surface proteins expressed during several different lifecycle stages (Table 1). Sequences were collected from GenBank and further sequences or haplotypes were reconstructed from published data. If only the haplotypes and frequencies were available the appropriate number of copies for each allele was added to the dataset to ensure natural population frequencies (Table S1). Additional sequence data from cultured or field isolates not fitting the above criteria were also collected, including those upon which vaccines have been based (Table S2). These sequences were included in the calculation of the (known) extent of diversity worldwide (Table 1) and in the cluster analyses to maximize the sample number and provide a reference for vaccine development. Tables S1 and S2 contain summary information (e.g. GenBank accession numbers, reference) for each of these dataset. The sequences and haplotypes are available from the authors upon request. For *msp1* and *msp2* all DNA sequences were translated using TranSeq (http://www.ebi.ac.uk/Tools/emboss/transeq/). For simple multiple alignments with few gaps, DNA sequences were aligned using Sequencher 4.8 (Gene Codes, Ann Arbor, MI). Amino acid alignments (MSP1 and MSP2) were done using Clustal W [62]. Gaps were removed from all alignments because indels and repeats evolve by different mechanisms to SNPs and may result in false estimates of biologically significant diversity. We also removed invariant sites and synonymous SNPs to simplify the haplotype and focus the analysis only on the putative antigenic diversity. The resultant nonsynonymous SNP (nsSNP) haplotypes or polymorphic amino acid haplotypes (for MSP1 and MSP2) were then used for population genetic analysis.

### Population Genetics

Population genetic analyses were first done with the complete dataset (Tables S1 and S2) to investigate the global range of diversity as well as the frequency of haplotypes being used in vaccine development, while the population dataset (Table S1) was used to investigate the range and distribution of diversity within and among the natural *P. falciparum* populations of individual countries. Population genetic parameters were determined using DnaSP v. 4.20.2 [63]. However, for MSP1 and MSP2 amino acid sequences we used Arlequin v. 3.1.1 [64] because DnaSP only handles DNA sequences. As measures of diversity we defined the *polymorphism* by counting the total number of synonymous (*dS*) and number of nonsynonymous (*dN*) SNPs; and by calculating from nsSNP haplotypes, the number of polymorphic sites (*S*), the average pairwise number of polymorphisms (*k*) and from complete DNA sequences (minus any gaps) the nucleotide diversity (*∏*), the latter being a proportional measure of polymorphism that can be compared among antigens. Additional measures of *diversity* calculated included the number of distinct haplotypes (*h*) (although this is heavily biased by sample size) and the *haplotype diversity* which is analogous to the heterozygosity (*Hd* = [n/(n−1)][(1−Σ(*f_i_*)^2^)] where n is the sample size and *f* is the frequency of the *i^t^* ^h^ allele) and can also be compared among antigens. The Mann-Whitney test was used to compare polymorphism or diversity among regions where at least 3 countries were included per region (or subregion). Spearman's rank correlation coefficient (*ρ*) was used to measure associations between polymorphism (*Π*) and diversity (*Hd*). Statistical analysis was done using SPSS v. 17.

To assess population structure we first estimated the *genetic differentiation* (i.e. the difference in the average diversity within compared to that among populations) for each antigen by calculating *F*_ST_ from both haplotype frequencies and pairwise sequence diversity. For comparisons among countries or regions for all antigens except MSP1, two transformed *F*_ST_ statistics available in DnaSP, were calculated namely *H*_ST_ which is loosely based on *Hd*, and *K*_ST_* based on *k*. Importantly, both *H*_ST_ and *K*_ST_* are weighted for variable population size [25]. Significance was tested by comparison with 95% confidence intervals from 1000 permutations [25]. For comparisons among defined natural populations within a country (i.e. >8 sequences in each) we calculated Weir and Cockerhams θ [64] from the pairwise sequence diversity (i.e. analogous to *K*_ST_*) in Arlequin. For MSP1 we measured the equivalent to *H*_ST_ available in Arlequin software, namely Weir and Cockerhams θ [64] calculated from haplotype frequencies. Significance was tested by the permutation test. *Gene flow* (*Nm*) was calculated using the method of Hudson *et al.* [65]. Population structure was also assessed using the *Bayesian clustering* algorithm implemented in *structure* v. 2.2 [27], [36], which assigns individual multi-locus genotypes probabilistically to a user-defined number of clusters (*K*) [27]. For each set of antigen haplotypes, *structure* was run 20 times for *K* = 1–10 for 10,000 Monte Carlo Markov Chain (MCMC) iterations after a burn-in period of 10,000 [66] using the admixture model and correlated allele frequencies. The mean log probability of the data (Ln*P*[D]) and its standard deviation was plotted to predict the optimal value for *K*. Membership coefficients (*Q*) were then averaged across individuals within countries and/or regions to reveal any geographic association of the resultant clusters. To quantify the distribution of clusters within a geographically defined region we developed a *population diversity* statistic, *Pd*, where *Pd* = 1−∑(*f*_i_)^2^, where *f*_i_ is the frequency of the i^th^ cluster (analogous to *Hd*). A low *Pd* (<0.5) indicated that the geographically defined population (e.g.. country, village) has parasites with predominant membership to one cluster, and high *Pd* (>0.5) indicated membership to multiple clusters with low-medium frequencies. We confirmed the cluster analysis by visualizing the relationships between isolates using a transparent *network analysis* technique which simply connects isolates, represented as nodes within a network, based on shared SNPs. Unlike phylogenetic methods there is no evolutionary model behind network construction, but a simple threshold was used to define where an edge was drawn. For each antigen, this threshold was defined so as best to visualize the relationships between isolates, and in particular the recombinant isolates. The software program R and the ‘network’ package was used to construct and visualize the antigen networks [67], [68].

## Supporting Information

Text S1 Haplotype-frequency vs. sequence based F-statistics and supporting references
(0.13 MB DOC)

Figure S1 Log probability of the data plots for Bayesian cluster analysis. LnP(D) is shown for nsSNP haplotypes of (A) *csp*, (B) *trap*, (C) *lsa1*, (D) *ama1*, (E) *eba175*, (F) *msp1*, (G) *msp3*, (H) *msp4*, (I) *glurp* and (J) *pfs48/45*. A plot of the log probability of the data, LnP(D) against all estimates of the number of clusters, *K*, was used to estimate the true value of *K*. LnP(D) typically plateaus or continues to increase slightly when true *K* has been reached (68). The error bars represent the mean value of 20 replicate runs at each *K* value. For some antigens, LnP(D) did not plateau with increasing *K*, in which case the lowest value that captured the major structure in the data was chosen (69).
(0.36 MB TIF)

Figure S2 Bayesian cluster analysis of nsSNP haplotypes for optimum K ± 1. Note the excess of admixed individuals for *K*+1. Subgroups: Dark blue = 1; Red = 2; Green = 3; Purple = 4; Light blue = POP5; Orange = 6.
(1.48 MB PDF)

Figure S3 Comparison of Bayesian cluster and network analysis for ten *P. falciparum* vaccine antigen genes. Subgroups: Dark blue = 1; Red = 2; Green = 3; Purple = 4; Light blue = 5; Orange = 6.
(0.66 MB TIF)

Figure S4 Local population structure for *P. falciparum* vaccine antigens based on Bayesian cluster analysis. Comparison of Bayesian cluster and network analysis for ten *P. falciparum* vaccine antigen genes. Networks (as shown in Figure 3) are shown with individuals shaded by the *structure*-defined subgroups (as shown in Figure 2). Subgroups: Dark blue = 1; Red = 2; Green = 3; Purple = 4; Light blue = 5; Orange = 6; Admixed haplotypes (those having <75% membership to any one cluster) are shown in white, vaccine haplotypes are shown in yellow.
(0.90 MB TIF)

Table S1 Population dataset for twelve *P. falciparum* vaccine antigen genes.
(0.04 MB XLS)

Table S2 Dataset 2: Sequences from laboratory and other isolates for twelve *P. falciparum* vaccine antigen genes.
(0.02 MB XLS)

Table S3 Analysis of *structure* results for ten *P. falciparum* vaccine antigens.
(0.02 MB XLS)

Table S4 Haplotype and cluster membership for laboratory isolates.
(0.03 MB XLS)
