# Supplementary material for: Contrasting Population Structures of the Genes Encoding Ten Leading Vaccine-Candidate Antigens of the Human Malaria Parasite, Plasmodium falciparum
Source: PLoS One. 2009 Dec 30;4(12):e8497. doi: 10.1371/journal.pone.0008497 (PMC2795866; doi:10.1371/journal.pone.0008497)
Supplement: Text S1 — Haplotype-frequency vs. sequence based F-statistics and supporting references (0.13 MB DOC) [file pone.0008497.s001.doc]

**TEXT S1.**

**Haplotype frequency vs. sequence based *F*-statistics**

Haplotype frequency based *F*_ST_ statistics consider all haplotypes to be equidistant and simply measure differences in the frequencies of haplotypes among populations. Sequence diversity based *F*_ST_ statistics consider the range of polymorphism among pairs of haplotypes and differential weighting is therefore applied to comparisons. For high diversity genes, such as those encoding *P. falciparum* antigens the sequence diversity statistics will be more sensitive because they consider the full range of diversity, whereas the differences between the two statistics will be negligible for lower diversity genes with a narrower range of diversity. This differential sensitivity was observed for *trap*, the most diverse antigen where significant geographic population structure was observed in the Asia Pacific using sequence diversity but not using haplotype frequencies (Table 3). The opposite was shown for *lsa1* with sequence diversity statistics demonstrating a slightly lower degree of structuring in the Asia Pacific than did those using haplotype frequencies, indicating that for this antigen differences were exaggerated by the latter statistics (Table 3). Haplotype frequency-based analysis has also been shown to perform better than that for sequence diversity with smaller sample sizes [70] and may be a better estimate of population structure for those antigens for which sample size was limiting (i.e. *lsa1, glurp, pfs48/45*). Thus sequence diversity will give the most accurate estimate of population structure for the majority of the antigen genes but haplotype frequency analysis is also informative.

**Supporting References**

1. de Stricker K, Vuust J, Jepsen S, Oeuvray C, & Theisen M (2000) Conservation and heterogeneity of the glutamate-rich protein (GLURP) among field isolates and laboratory lines of Plasmodium falciparum. *Mol Biochem Parasitol* 111(1):123-130 .

2. Yoshida N*, et al.* (1990) Plasmodium falciparum: restricted polymorphism of T cell epitopes of the circumsporozoite protein in Brazil. *Exp Parasitol* 71(4):386-392 .

3. Escalante AA*, et al.* (2002) A study of genetic diversity in the gene encoding the circumsporozoite protein (CSP) of Plasmodium falciparum from different transmission areas--XVI. Asembo Bay Cohort Project. *Mol Biochem Parasitol* 125(1-2):83-90 .

4. Tanabe K, Sakihama N, & Kaneko A (2004) Stable SNPs in malaria antigen genes in isolated populations. *Science* 303(5657):493 .

5. Jalloh A*, et al.* (2006) Sequence variation in the T-cell epitopes of the Plasmodium falciparum circumsporozoite protein among field isolates is temporally stable: a 5-year longitudinal study in southern Vietnam. *J Clin Microbiol* 44(4):1229-1235.

6. Jongwutiwes S, Tanabe K, Hughes MK, Kanbara H, & Hughes AL (1994) Allelic variation in the circumsporozoite protein of Plasmodium falciparum from Thai field isolates. *Am J Trop Med Hyg* 51(5):659-668 .

7. Zakeri S, Avazalipoor M, Mehrizi AA, Djadid ND, & Snounou G (2007) Restricted T-cell epitope diversity in the circumsporozoite protein from Plasmodium falciparum populations prevalent in Iran. *Am J Trop Med Hyg* 76(6):1046-1051 .

8. Weedall GD, Preston BM, Thomas AW, Sutherland CJ, & Conway DJ (2007) Differential evidence of natural selection on two leading sporozoite stage malaria vaccine candidate antigens. *Int J Parasitol* 37(1):77-85.

9. Jongwutiwes S, Putaporntip C, Kanbara H, & Tanabe K (1998) Variation in the thrombospondin-related adhesive protein (TRAP) gene of Plasmodium falciparum from Thai field isolates. *Mol Biochem Parasitol* 92(2):349-353 .

10. Yang C*, et al.* (1995) Sequence variations in the non-repetitive regions of the liver stage-specific antigen-1 (LSA-1) of Plasmodium falciparum from field isolates. *Mol Biochem Parasitol* 71(2):291-294.

11. Ravichandran M, Doolan DL, Cox-Singh J, Hoffman SL, & Singh B (2000) Research note: HLA degenerate T-cell epitopes from Plasmodium falciparum liver stage-specific antigen 1 (LSA-1) are highly conserved in isolates from geographically distinct areas. *Parasite Immunol* 22(9):469-473 .

12. Escalante AA*, et al.* (2001) Polymorphism in the gene encoding the apical membrane antigen-1 (AMA-1) of Plasmodium falciparum. X. Asembo Bay Cohort Project. *Mol Biochem Parasitol* 113(2):279-287 .

13. Cortes A*, et al.* (2003) Geographical structure of diversity and differences between symptomatic and asymptomatic infections for Plasmodium falciparum vaccine candidate AMA1. *Infect Immun* 71(3):1416-1426.

14. Polley SD, Chokejindachai W, & Conway DJ (2003) Allele frequency-based analyses robustly map sequence sites under balancing selection in a malaria vaccine candidate antigen. *Genetics* 165(2):555-561 .

15. Rajesh V*, et al.* (2008) Plasmodium falciparum: genetic polymorphism in apical membrane antigen-1 gene from Indian isolates. *Exp Parasitol* 119(1):144-151 .

16. Garg S*, et al.* (2007) Sequence diversity and natural selection at domain I of the apical membrane antigen 1 among Indian Plasmodium falciparum populations. *Malar J* 6:154 .

17. Polley SD & Conway DJ (2001) Strong diversifying selection on domains of the Plasmodium falciparum apical membrane antigen 1 gene. *Genetics* 158(4):1505-1512.

18. Duan J*, et al.* (2008) Population structure of the genes encoding the polymorphic Plasmodium falciparum apical membrane antigen 1: implications for vaccine design. *Proc Natl Acad Sci U S A* 105(22):7857-7862 .

19. Kocken CH*, et al.* (2000) Molecular characterisation of Plasmodium reichenowi apical membrane antigen-1 (AMA-1), comparison with P. falciparum AMA-1, and antibody-mediated inhibition of red cell invasion. *Mol Biochem Parasitol* 109(2):147-156 .

20. Verra F*, et al.* (2006) Contrasting signatures of selection on the Plasmodium falciparum erythrocyte binding antigen gene family. *Mol Biochem Parasitol* 149(2):182-190 .

21. Baum J, Thomas AW, & Conway DJ (2003) Evidence for diversifying selection on erythrocyte-binding antigens of Plasmodium falciparum and P. vivax. *Genetics* 163(4):1327-1336 .

22. Da Silveira LA*, et al.* (1999) Allelic diversity and antibody recognition of Plasmodium falciparum merozoite surface protein 1 during hypoendemic malaria transmission in the Brazilian amazon region. *Infect Immun* 67(11):5906-5916 .

23. Chenet SM, Branch OH, Escalante AA, Lucas CM, & Bacon DJ (2008) Genetic diversity of vaccine candidate antigens in Plasmodium falciparum isolates from the Amazon basin of Peru. *Malar J* 7:93 .

24. Sakihama N*, et al.* (2006) Limited allelic diversity of Plasmodium falciparum merozoite surface protein 1 gene from populations in the Solomon Islands. *Am J Trop Med Hyg* 74(1):31-40 .

25. Sakihama N, Kaneko A, Hattori T, & Tanabe K (2001) Limited recombination events in merozoite surface protein-1 alleles of Plasmodium falciparum on islands. *Gene* 279(1):41-48 .

26. Sakihama N*, et al.* (2007) Allelic diversity in the merozoite surface protein 1 gene of Plasmodium falciparum on Palawan Island, the Philippines. *Parasitol Int* 56(3):185-194 .

27. Kaneko O, Kimura M, Kawamoto F, Ferreira MU, & Tanabe K (1997) Plasmodium falciparum: allelic variation in the merozoite surface protein 1 gene in wild isolates from southern Vietnam. *Exp Parasitol* 86(1):45-57 .

28. Ferreira MU, Ribeiro WL, Tonon AP, Kawamoto F, & Rich SM (2003) Sequence diversity and evolution of the malaria vaccine candidate merozoite surface protein-1 (MSP-1) of Plasmodium falciparum. *Gene* 304:65-75 .

29. Sakihama N*, et al.* (1999) Allelic recombination and linkage disequilibrium within Msp-1 of Plasmodium falciparum, the malignant human malaria parasite. *Gene* 230(1):47-54 .

30. Lalitha PV, Malhotra P, Chattopadhyay R, & Chauhan VS (1999) Plasmodium falciparum: variations in the C-terminal cysteine-rich region of the merozoite surface protein-1 in field samples among Indian isolates. *Exp Parasitol* 92(1):12-18 .

31. Vijay Kumar S*, et al.* (2005) Plasmodium falciparum: genetic diversity of C-terminal region of MSP-1 in isolates from Indian sub-continent. *Exp Parasitol* 110(4):384-388 .

32. Mamillapalli A*, et al.* (2007) Polymorphism and epitope sharing between the alleles of merozoite surface protein-1 of Plasmodium falciparum among Indian isolates. *Malar J* 6:95 .

33. Mehrizi AA, Zakeri S, Salmanian AH, Sanati MH, & Djadid ND (2008) Plasmodium falciparum: sequence analysis of the gene encoding the C-terminus region of the merozoite surface protein-1, a potential malaria vaccine antigen, in Iranian clinical isolates. *Exp Parasitol* 118(3):378-385 .

34. Qari SH*, et al.* (1998) Predicted and observed alleles of Plasmodium falciparum merozoite surface protein-1 (MSP-1), a potential malaria vaccine antigen. *Mol Biochem Parasitol* 92(2):241-252 .

35. Takala SL*, et al.* (2007) Dynamics of Polymorphism in a Malaria Vaccine Antigen at a Vaccine-Testing Site in Mali. *PLoS Med* 4(3):e93.

36. Sallenave-Sales S*, et al.* (2003) Plasmodium falciparum: limited genetic diversity of MSP-2 in isolates circulating in Brazilian endemic areas. *Exp Parasitol* 103(3-4):127-135 .

37. Tonon AP*, et al.* (2004) Plasmodium falciparum: sequence diversity and antibody recognition of the Merozoite surface protein-2 (MSP-2) in Brazilian Amazonia. *Exp Parasitol* 108(3-4):114-125 .

38. Eisen D, Billman-Jacobe H, Marshall VF, Fryauff D, & Coppel RL (1998) Temporal variation of the merozoite surface protein-2 gene of Plasmodium falciparum. *Infect Immun* 66(1):239-246 .

39. Weisman S*, et al.* (2001) Antibody responses to infections with strains of Plasmodium falciparum expressing diverse forms of merozoite surface protein 2. *Infect Immun* 69(2):959-967 .

40. Polley SD*, et al.* (2007) Plasmodium falciparum merozoite surface protein 3 is a target of allele-specific immunity and alleles are maintained by natural selection. *J Infect Dis* 195(2):279-287 .

41. Benet A, Tavul L, Reeder JC, & Cortes A (2004) Diversity of the Plasmodium falciparum vaccine candidate merozoite surface protein 4 (MSP4) in a natural population. *Mol Biochem Parasitol* 134(2):275-280.

42. Polson HE, Conway DJ, Fandeur T, Mercereau-Puijalon O, & Longacre S (2005) Gene polymorphism of Plasmodium falciparum merozoite surface proteins 4 and 5. *Mol Biochem Parasitol* 142(1):110-115 .

43. Jongwutiwes S, Putaporntip C, Friedman R, & Hughes AL (2002) The extent of nucleotide polymorphism is highly variable across a 3-kb region on Plasmodium falciparum chromosome 2. *Mol Biol Evol* 19(9):1585-1590 .

44. Escalante AA*, et al.* (2002) Polymorphism in the gene encoding the Pfs48/45 antigen of Plasmodium falciparum. XI. Asembo Bay Cohort Project. *Mol Biochem Parasitol* 119(1):17-22 .

45. Gardner MJ*, et al.* (2002) Genome sequence of the human malaria parasite Plasmodium falciparum. *Nature* 419(6906):498-511.

46. del Portillo HA, Nussenzweig RS, & Enea V (1987) Circumsporozoite gene of a Plasmodium falciparum strain from Thailand. *Mol Biochem Parasitol* 24(3):289-294 .

47. Lockyer MJ & Schwarz RT (1987) Strain variation in the circumsporozoite protein gene of Plasmodium falciparum. *Mol Biochem Parasitol* 22(1):101-108 .

48. Robson KJ*, et al.* (1990) Polymorphism of the TRAP gene of Plasmodium falciparum. *Proc Biol Sci* 242(1305):205-216 .

49. Robson KJ*, et al.* (1998) Natural polymorphism in the thrombospondin-related adhesive protein of Plasmodium falciparum. *Am J Trop Med Hyg* 58(1):81-89 .

50. Fidock DA*, et al.* (1994) Plasmodium falciparum liver stage antigen-1 is well conserved and contains potent B and T cell determinants. *J Immunol* 153(1):190-204 .

51. Marshall VM, Zhang L, Anders RF, & Coppel RL (1996) Diversity of the vaccine candidate AMA-1 of Plasmodium falciparum. *Mol Biochem Parasitol* 77(1):109-113 .

52. Liang H & Sim BK (1997) Conservation of structure and function of the erythrocyte-binding domain of Plasmodium falciparum EBA-175. *Mol Biochem Parasitol* 84(2):241-245 .

53. Sim BK (1990) Sequence conservation of a functional domain of erythrocyte binding antigen 175 in Plasmodium falciparum. *Mol Biochem Parasitol* 41(2):293-295 .

54. Ware LA*, et al.* (1993) Two alleles of the 175-kilodalton Plasmodium falciparum erythrocyte binding antigen. *Mol Biochem Parasitol* 60(1):105-109 .

55. Jongwutiwes S, Tanabe K, & Kanbara H (1993) Sequence conservation in the C-terminal part of the precursor to the major merozoite surface proteins (MSP1) of Plasmodium falciparum from field isolates. *Mol Biochem Parasitol* 59(1):95-100 .

56. Tolle R, Bujard H, & Cooper JA (1995) Plasmodium falciparum: variations within the C-terminal region of merozoite surface antigen-1. *Exp Parasitol* 81(1):47-54 .

57. Pan W, Tolle R, & Bujard H (1995) A direct and rapid sequencing strategy for the Plasmodium falciparum antigen gene gp190/MSA1. *Mol Biochem Parasitol* 73(1-2):241-244 .

58. Chang SP*, et al.* (1988) Plasmodium falciparum: gene structure and hydropathy profile of the major merozoite surface antigen (gp195) of the Uganda-Palo Alto isolate. *Exp Parasitol* 67(1):1-11 .

59. Holder AA*, et al.* (1985) Primary structure of the precursor to the three major surface antigens of Plasmodium falciparum merozoites. *Nature* 317(6034):270-273 .

60. Kang Y & Long CA (1995) Sequence heterogeneity of the C-terminal, Cys-rich region of the merozoite surface protein-1 (MSP-1) in field samples of Plasmodium falciparum. *Mol Biochem Parasitol* 73(1-2):103-110 .

61. Huber W*, et al.* (1997) Limited sequence polymorphism in the Plasmodium falciparum merozoite surface protein 3. *Mol Biochem Parasitol* 87(2):231-234 .

62. McColl DJ & Anders RF (1997) Conservation of structural motifs and antigenic diversity in the Plasmodium falciparum merozoite surface protein-3 (MSP-3). *Mol Biochem Parasitol* 90(1):21-31 .

63. Wang L, Marshall VM, & Coppel RL (2002) Limited polymorphism of the vaccine candidate merozoite surface protein 4 of Plasmodium falciparum. *Mol Biochem Parasitol* 120(2):301-303 .

64. Wu T, Black CG, Wang L, Hibbs AR, & Coppel RL (1999) Lack of sequence diversity in the gene encoding merozoite surface protein 5 of Plasmodium falciparum. *Mol Biochem Parasitol* 103(2):243-250 .

65. Borre MB*, et al.* (1991) Primary structure and localization of a conserved immunogenic Plasmodium falciparum glutamate rich protein (GLURP) expressed in both the preerythrocytic and erythrocytic stages of the vertebrate life cycle. *Mol Biochem Parasitol* 49(1):119-131 .

66. Anthony TG, Polley SD, Vogler AP, & Conway DJ (2007) Evidence of non-neutral polymorphism in Plasmodium falciparum gamete surface protein genes Pfs47 and Pfs48/45. *Mol Biochem Parasitol* 156(2):117-123 .

67. Kocken CH*, et al.* (1995) Minimal variation in the transmission-blocking vaccine candidate Pfs48/45 of the human malaria parasite Plasmodium falciparum. *Mol Biochem Parasitol* 69(1):115-118 .

68. Evanno G, Regnaut S, & Goudet J (2005) Detecting the number of clusters of individuals using the software STRUCTURE: a simulation study. *Mol Ecol* 14(8):2611-2620.

69. Pritchard JK, Wen, X and Falush, D. (2007) Documentation for *structure* software: Version 2.3. <http://pritch.bsd.uchicago.edu/structure.html)>.

70. Hudson RR, Boos DD, Kaplan NL (1992) A statistical test for detecting geographic subdivision. *Mol Biol Evol* 9: 138-151.
