# Supplementary figures and images for: Contrasting Population Structures of the Genes Encoding Ten Leading Vaccine-Candidate Antigens of the Human Malaria Parasite, Plasmodium falciparum
Source: PLoS One. 2009 Dec 30;4(12):e8497. doi: 10.1371/journal.pone.0008497 (PMC2795866; doi:10.1371/journal.pone.0008497)

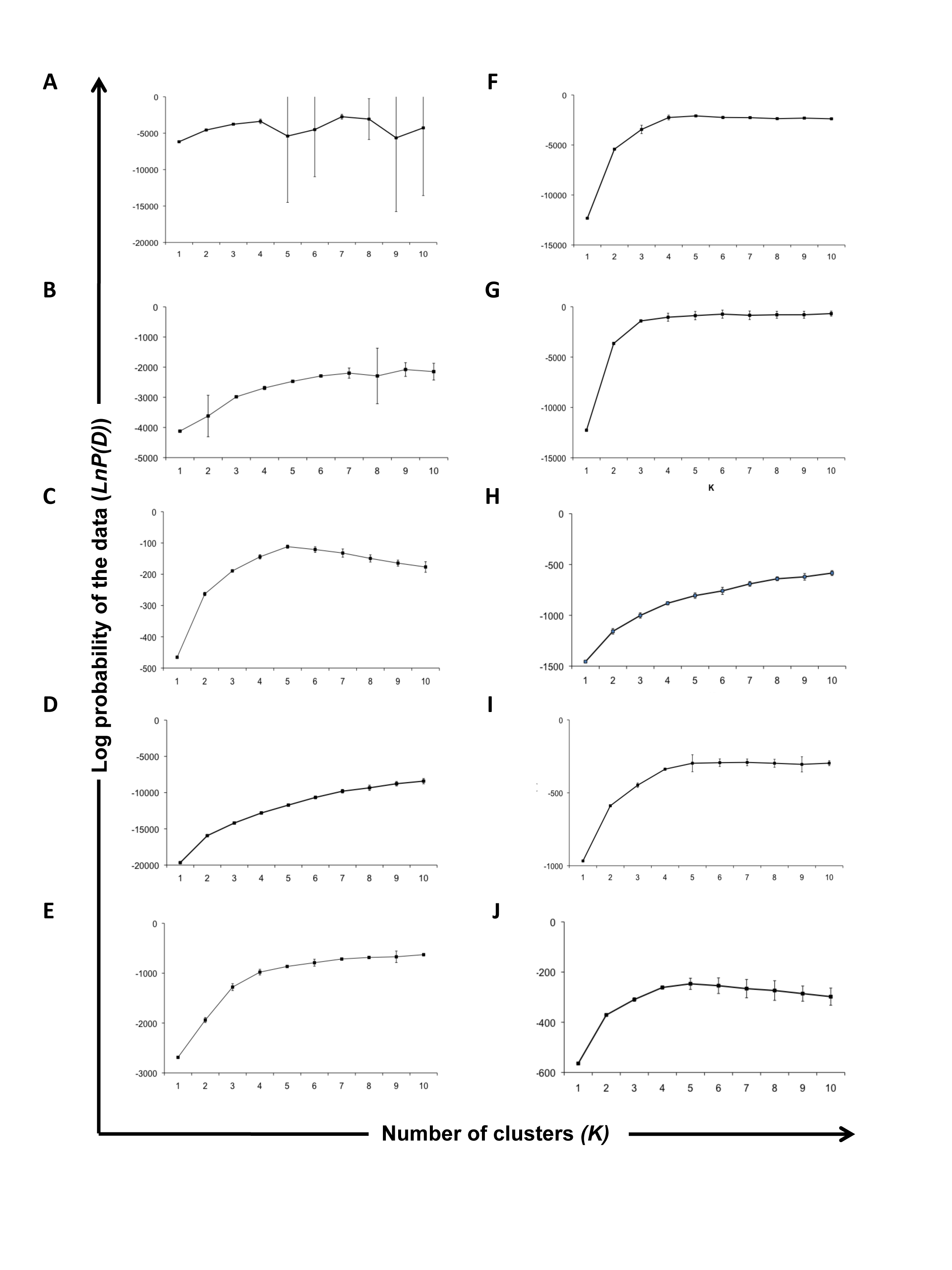

Supplement: Figure S1 — Log probability of the data plots for Bayesian cluster analysis. LnP(D) is shown for nsSNP haplotypes of (A) csp, (B) trap, (C) lsa1, (D) ama1, (E) eba175, (F) msp1, (G) msp3, (H) msp4, (I) glurp and (J) pfs48/45. A plot of the log probability of the data, LnP(D) against all estimates of the number of clusters, K, was used to estimate the true value of K. LnP(D) typically plateaus or continues to increase slightly when true K has been reached (68). The error bars represent the mean value of 20 replicate runs at each K value. For some antigens, LnP(D) did not plateau with increasing K, in which case the lowest value that captured the major structure in the data was chosen (69). (0.36 MB TIF) [file pone.0008497.s002.tif]

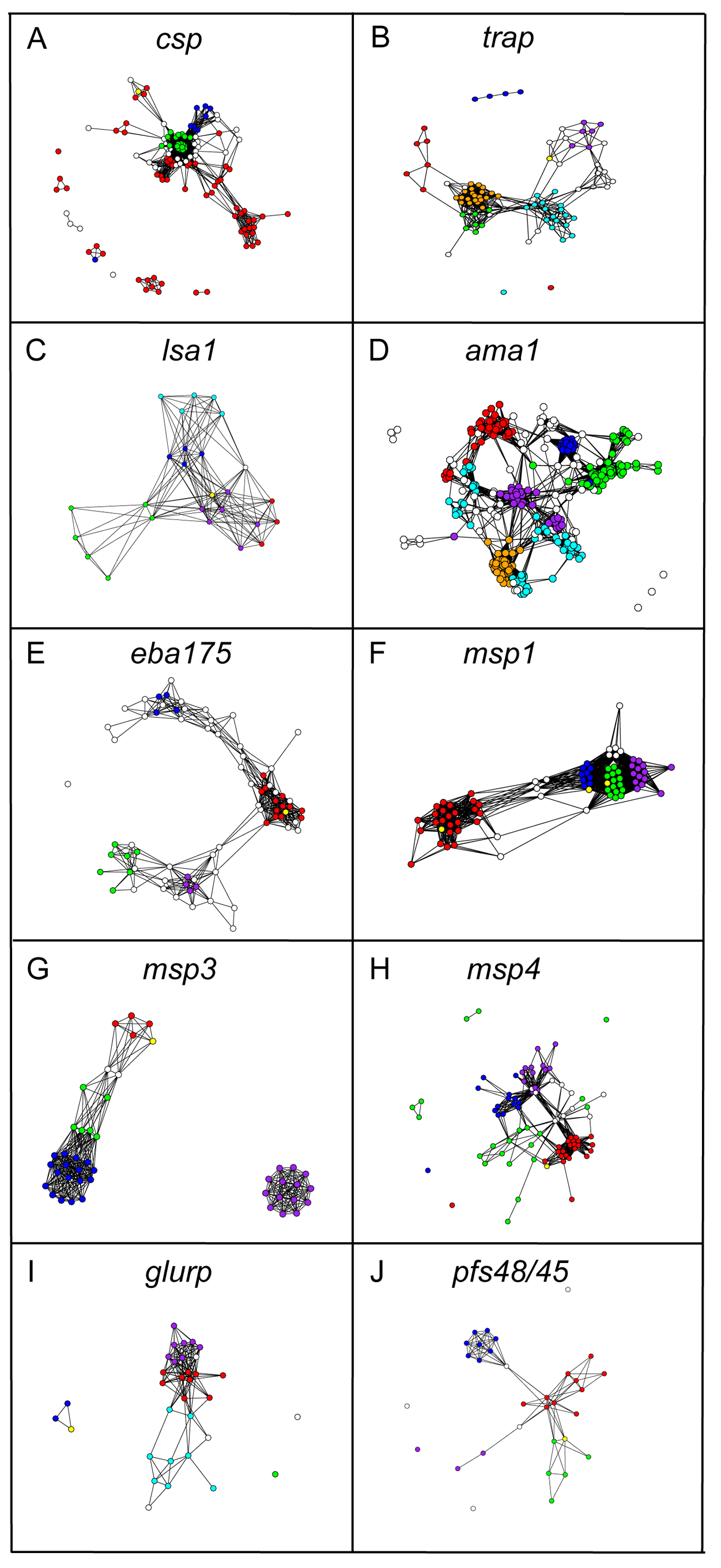

Supplement: Figure S3 — Comparison of Bayesian cluster and network analysis for ten P. falciparum vaccine antigen genes. Subgroups: Dark blue = 1; Red = 2; Green = 3; Purple = 4; Light blue = 5; Orange = 6. (0.66 MB TIF) [file pone.0008497.s004.tif]

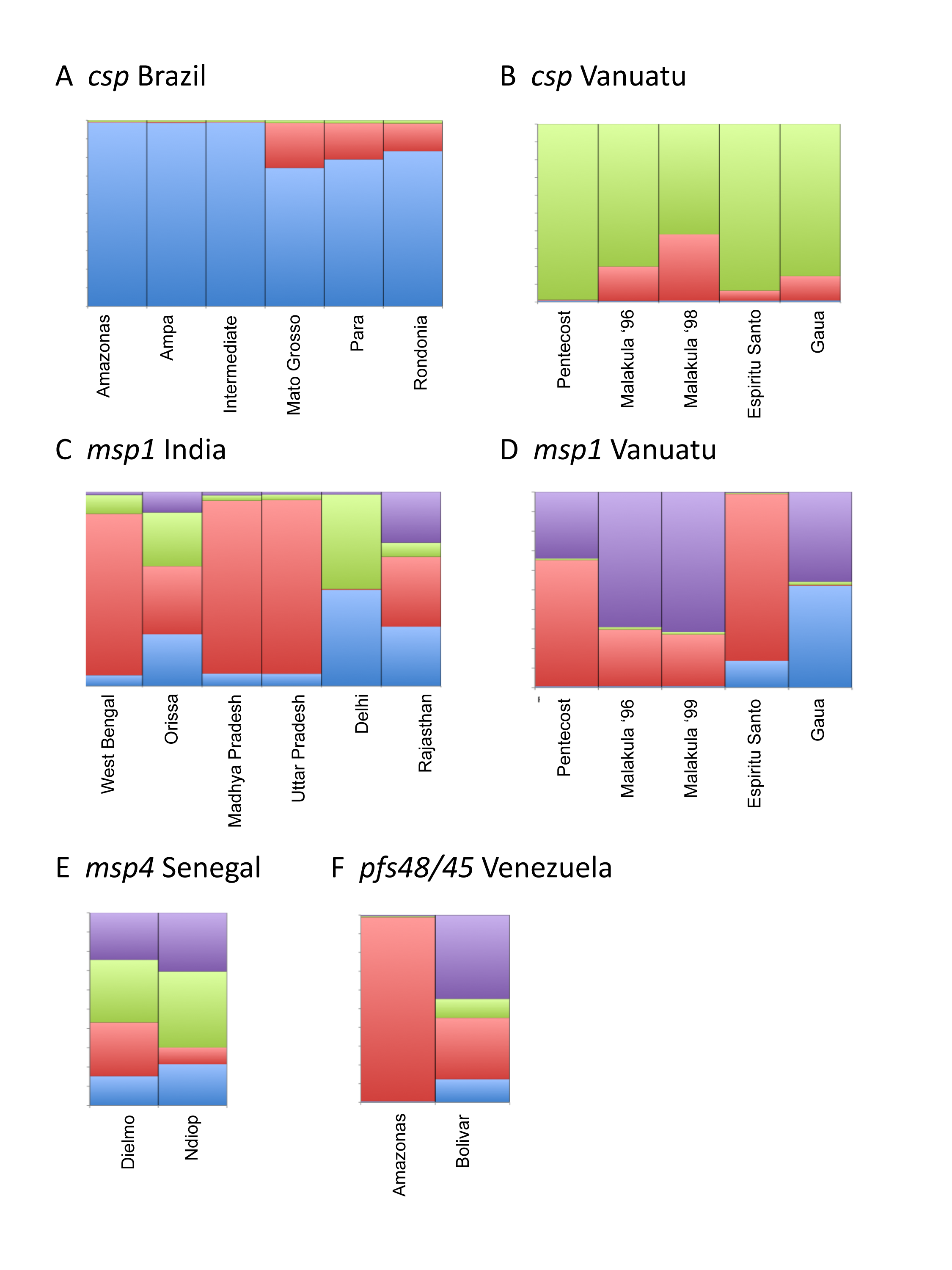

Supplement: Figure S4 — Local population structure for P. falciparum vaccine antigens based on Bayesian cluster analysis. Comparison of Bayesian cluster and network analysis for ten P. falciparum vaccine antigen genes. Networks (as shown in Figure 3) are shown with individuals shaded by the structure-defined subgroups (as shown in Figure 2). Subgroups: Dark blue = 1; Red = 2; Green = 3; Purple = 4; Light blue = 5; Orange = 6; Admixed haplotypes (those having <75% membership to any one cluster) are shown in white, vaccine haplotypes are shown in yellow. (0.90 MB TIF) [file pone.0008497.s005.tif]
