# Supplementary material for: Contrasting Population Structures of the Genes Encoding Ten Leading Vaccine-Candidate Antigens of the Human Malaria Parasite, Plasmodium falciparum
Source: PLoS One. 2009 Dec 30;4(12):e8497. doi: 10.1371/journal.pone.0008497 (PMC2795866; doi:10.1371/journal.pone.0008497)
Supplement: Figure S2 — Bayesian cluster analysis of nsSNP haplotypes for optimum K ± 1. Note the excess of admixed individuals for K+1. Subgroups: Dark blue = 1; Red = 2; Green = 3; Purple = 4; Light blue = POP5; Orange = 6. (1.48 MB PDF) [file pone.0008497.s003.pdf]

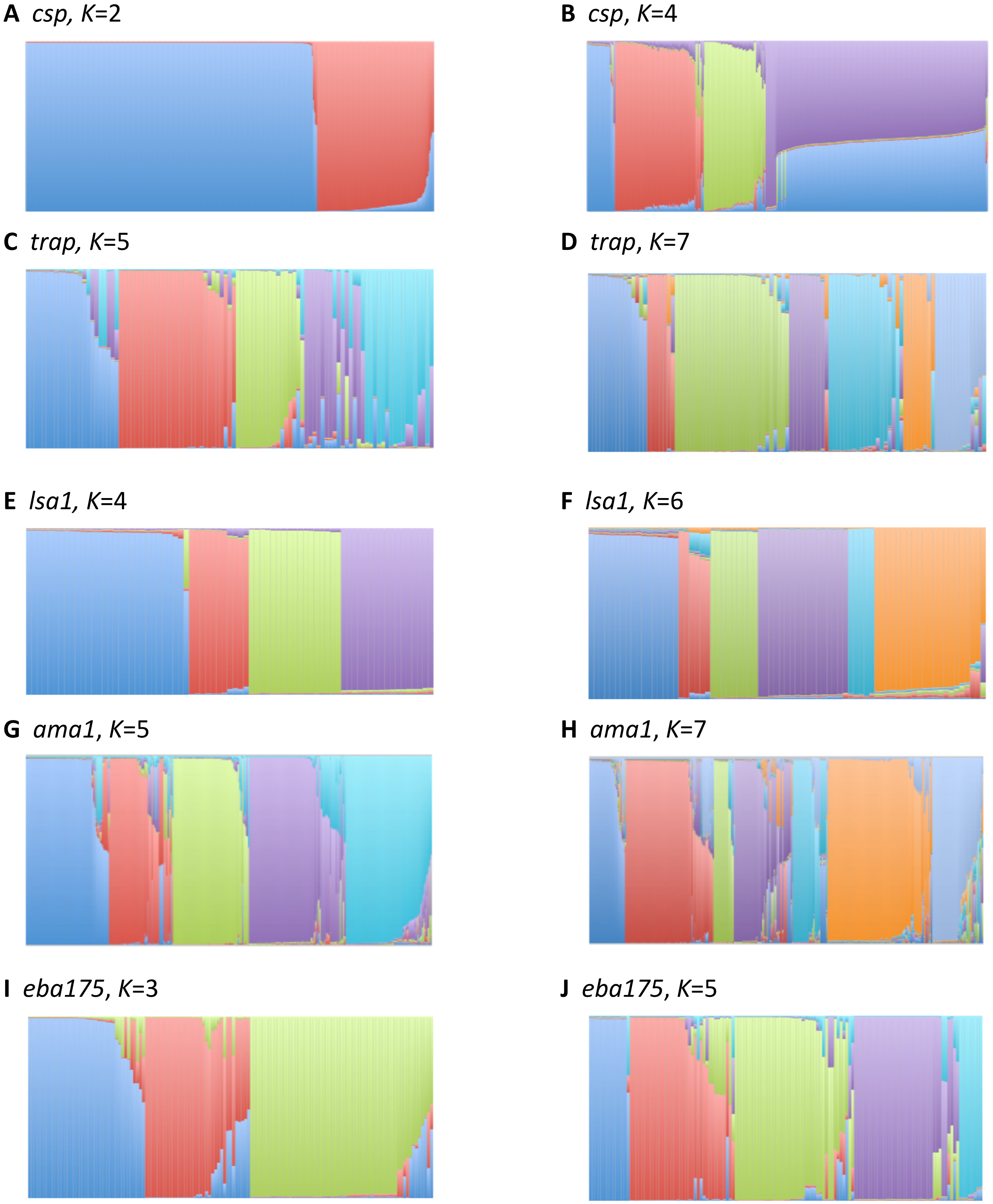
A csp, K=2
B csp, K=4
C trap, K=5
D trap, K=7
E lsa1, K=4
F lsa1, K=6
G ama1, K=5
H ama1, K=7
I eba175, K=3
J eba175, K=5

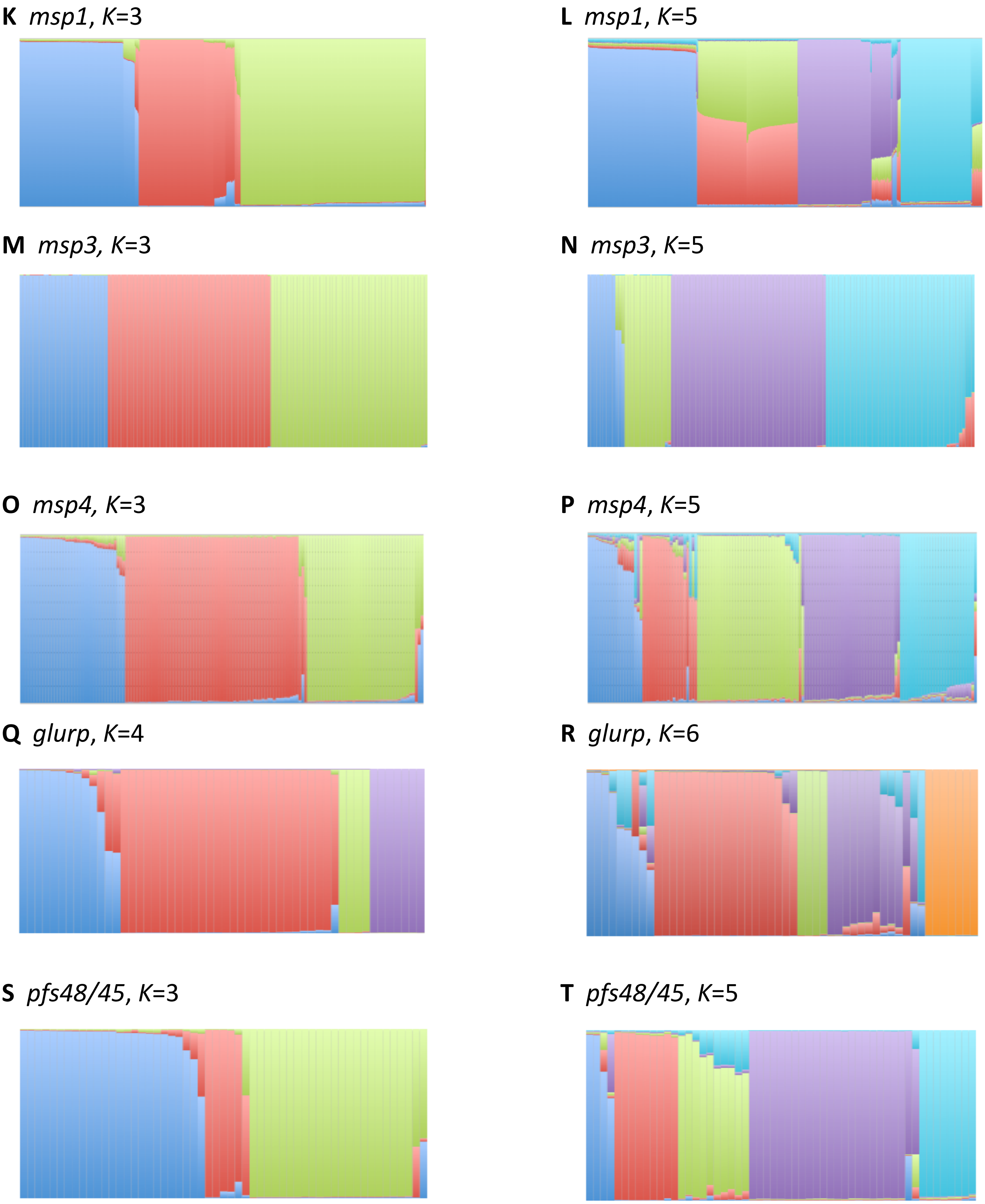

K msp1, K=3
L msp1, K=5
M msp3, K=3
N msp3, K=5
O msp4, K=3
P msp4, K=5
Q glurp, K=4
R glurp, K=6
S pfs48/45, K=3
T pfs48/45, K=5
